# Little devil takes your breath away

**DOI:** 10.1007/s12471-019-1284-8

**Published:** 2019-05-10

**Authors:** M. A. C. Koole, J. A. Winkelman, A. Kaya, M. A. Beijk

**Affiliations:** 1 Department of Cardiology, Red Cross Hospital, Beverwijk, The Netherlands; 2 Department of Cardiothoracic Surgery, Amsterdam UMC, University of Amsterdam, Amsterdam, The Netherlands; 3 Department of Cardiology, Amsterdam UMC, University of Amsterdam, Amsterdam, The Netherlands

## Answer

Neovascularisation of a large tumour in the left atrium.

**Fig. 1:**
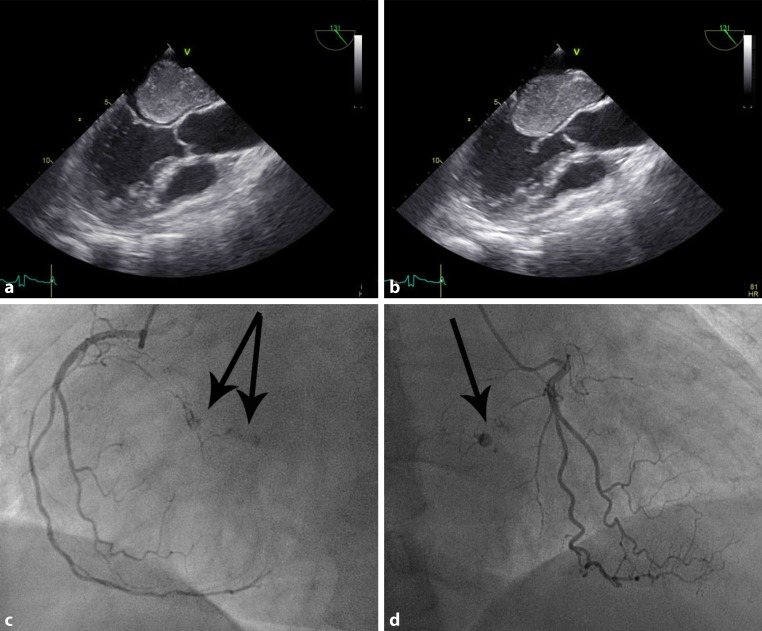
Transoesophageal echocardiographic imaging of left atrial myxoma **a** in systole and **b** in diastole, with prolapse of the tumour across the mitral valve. Coronary angiography of the right coronary artery (**c,** **d**) showing neovascularisation with a tumour blush (*arrows*)

The patient has a recent history of a transient ischaemic attack and atrial fibrillation treated with dabigatran. Recent chest radiography and spirometry test results were normal. Physical examination was unremarkable. Echocardiography revealed a large mobile mass in the left atrium attached to the atrial septum prolapsing across the mitral valve in diastole causing mitral valve obstruction (Fig. 1a, b). Preoperative coronary angiography showed neovascularisation with a tumour blush (Fig. 1c, d, arrows) arising from the right coronary artery. The tumour was surgically resected. Histological examination confirmed the diagnosis of a cardiac myxoma.

Left atrial myxoma is the most common primary cardiac tumour [1]. The clinical presentation is largely determined by the size, location and mobility of the tumour. Although cardiac myxomas are benign, the manifestations can be serious, i.e. embolisation, obstruction and arrhythmogenesis. Embolic manifestations may include stroke, myocardial infarction and/or visceral infarctions. Multimodality imaging is pivotal in diagnosing atrial myxomas. Preoperative coronary angiography may show the presence of vascularity of myxomas, mostly from the left circumflex artery [2]. This neovascularisation favours the diagnosis of a cardiac myxoma rather than a thrombus, which is usually non-vascularised [3]. Surgical resection should be performed as soon as possible after diagnosis, as the risk of embolisation is high. In cases with evidence of blood shunting, due to either spurting from the myxoma surface or fistula formation resulting in a steal phenomenon, these feeding vessels should be ligated during the surgery. Follow-up is mandatory for early detection of recurrence.
